# Supplementary material for: An Analysis of the Trend of Fetal Mortality Rates among Working and Jobless Households in Japan, 1995–2019
Source: Int J Environ Res Public Health. 2021 Apr 30;18(9):4810. doi: 10.3390/ijerph18094810 (PMC8125765; doi:10.3390/ijerph18094810)
Supplement: Supplementary file 1 [file ijerph-18-04810-s001.zip › ijerph-1177364-supplementary.pdf]

Table S1. The yearly artificial and spontaneous fetal mortality rates per 1000 births and GDP per capita in Japan.

Year	GDP per capita*	Crude rate		Maternal age-standardized rate	
		Artificial fetal mortality rate	Spontaneous fetal mortality rate	Artificial fetal mortality rate	Spontaneous fetal mortality rate
1995	4214.0	16.7	14.7	16.7	14.7
1996	4312.2	16.3	14.5	16.6	14.5
1997	4331.1	17.1	13.9	17.2	13.9
1998	4257.2	16.9	13.4	16.9	13.3
1999	4213.4	16.9	13.5	16.9	13.4
2000	4264.4	17.1	12.9	16.9	12.7
2001	4176.3	16.9	12.7	16.6	12.4
2002	4138.8	17.2	12.5	16.9	12.1
2003	4153.7	16.7	12.3	16.7	11.9
2004	4176.1	16.3	12.2	16.6	11.8
2005	4209.9	15.7	12.0	16.3	11.5
2006	4228.6	14.6	11.7	15.3	11.2
2007	4238.2	13.6	11.4	14.4	10.8
2008	4062.2	12.9	11.1	13.7	10.5
2009	3913.9	12.5	11.0	13.4	10.2
2010	3973.6	12.3	10.9	13.4	10.1
2011	3939.7	12.0	10.9	13.1	10.0
2012	3943.0	11.7	10.6	13.1	9.7
2013	3993.7	11.7	10.2	13.1	9.2
2014	4075.3	11.5	10.4	12.9	9.3
2015	4217.1	10.9	10.4	12.5	9.3
2016	4254.3	10.4	9.9	11.8	8.8
2017	4344.5	10.4	9.9	12.2	8.9
2018	4360.2	10.6	9.7	12.7	8.5
2019	4391.7	11.3	10.0	13.7	8.9

*Unit: thousand yen
